# Prosteatotic and Protective Components in a Unique Model of Fatty Liver: Gut Microbiota and Suppressed Complement System

**DOI:** 10.1038/srep31763

**Published:** 2016-08-23

**Authors:** Long Liu, Xing Zhao, Qian Wang, Xiaoxian Sun, Lili Xia, Qianqian Wang, Biao Yang, Yihui Zhang, Sean Montgomery, He Meng, Tuoyu Geng, Daoqing Gong

**Affiliations:** 1 College of Animal Science and Technology, Yangzhou University, Yangzhou 225009, China; 2 Department of Botany, University of British Columbia, 6270 University Boulevard, British Columbia, V6T 1Z4, Canada; 3 School of Agriculture and Biology, Shanghai Jiaotong University; Shanghai Key Laboratory of Veterinary Biotechnology, 800 Dongchuan Road, Shanghai 200240, China

## Abstract

Goose can develop severe hepatic steatosis without overt injury, thus it may serve as a unique model for uncovering how steatosis-related injury is prevented. To identify the markedly prosteatotic and protective mechanisms, we performed an integrated analysis of liver transcriptomes and gut microbial metagenomes using samples collected from overfed and normally-fed geese at different time points. The results indicated that the fatty liver transcriptome, initially featuring a ‘metabolism’ pathway, was later joined by ‘cell growth and death’ and ‘immune diseases’ pathways. Gut microbiota played a synergistic role in the liver response as microbial and hepatic genes affected by overfeeding shared multiple pathways. Remarkably, the complement system, an inflammatory component, was comprehensively suppressed in fatty liver, which was partially due to increased blood lactic acid from enriched *Lactobacillus*. Data from *in vitro* studies suggested that lactic acid suppressed TNFα via the HNF1α/C5 pathway. In conclusion, gut microbes and their hosts respond to excess energy influx as an organic whole, severe steatosis and related tolerance of goose liver may be partially attributable to gut microbiotic products and suppressed complement system, and lactic acid from gut microbiota participates in the suppression of hepatic TNFα/inflammation through the HNF1α/C5 pathway.

Fatty liver disease contains a sequence of clinical pathological syndromes from simple steatosis to steatohepatitis, fibrosis, and even cirrhosis. It can be divided into alcoholic fatty liver disease (AFLD) and non-alcoholic fatty liver disease (NAFLD) depending on alcohol intake^1^. The global and increasing prevalence of NAFLD constitutes a great threat to human health. Non-alcoholic steatohepatitis (NASH), characterized by inflammation, is a key step in the progression of NAFLD from simple steatosis to more advanced stages. A ‘two-hit’ hypothesis was previously proposed to illustrate the pathogenesis of NASH^2^, in which fat accumulation or simple steatosis in the liver represents the ‘first hit’, followed by the ‘second hit’ mainly from oxidative stress and increased cytokine release. Recently, hepatic steatosis is not only considered the ‘first hit’, but also an inducer of many distinct injurious factors^3^. Thus, the ‘two-hit’ hypothesis is now being modified into a ‘multiple parallel hits’ hypothesis^4^, in which insulin resistance (IR) is the first step, which in turn leads to hyperinsulinemia, hepatic steatosis due to increased *de novo* lipogenesis or influx of free fatty acids. Finally, hepatic injury due to oxidative and inflammatory stresses as well as enhanced lipid peroxidation occurs when the adaptive mechanism is overwhelmed^5^. In other words, although IR and increased fatty acid release from excessive visceral adiposity are responsible for the development of liver steatosis^6^, lipotoxicity and inflammation are the primary factors for liver damage or tolerance to severe steatosis. Indeed, patients with hepatic steatosis do not always develop necroinflammation or liver fibrosis^7^. This phenomenon is likely attributed to some unknown protective genetic or environmental factors that suppress lipotoxicity and inflammation. Identifying the protective factors may provide a preventative or therapeutic approach to dealing with the challenge that human and animal NAFLD poses.

Goose, as the descendant of a migratory bird, has an excellent capacity to deposit fat in the liver. In the goose industry, this capacity has been utilized for fatty liver (*foie gras*) production within 2–3 weeks of overfeeding. In overfed geese, liver weight can increase from approximately 100 g to approximately 800 g in 2 weeks due to a large amount of lipid accumulation^8^. The capacity for and the tolerance to severe hepatic steatosis without overt liver injuries^9^ suggests the existence of prosteatotic and protective components in goose. Using goose as a unique model for fatty liver study, these components may be identified and thus provide novel therapeutic targets to combat human NAFLD. So far, hepatic lipogenesis and lipid channeling have been investigated in the context of fatty liver induced by overfeeding^10^,^11^. With the emergence of next generation sequencing (NGS) techniques, many genes have been identified as associated with goose fatty liver^12^,^13^,^14^. A recent study suggests that the large amount of fat stored in goose liver results from an imbalance between the storage and secretion of exogenous and *de novo* synthesized endogenous lipids, as well as an absence of leptin gene homologs due to positive selection^15^. However, the mechanisms underlying the excellent capacity and tolerance of goose liver to severe steatosis still remain unclear.

In recent years, the effect of gut microbiota on NAFLD has become more highly appreciated. Le Roy *et al*.^16^ demonstrated that gut microbiota determines the development of NAFLD in mice^16^. Gut microbiota may contribute to NAFLD via their cellular components. The known gut-liver axis at least partially mediates the effect of gut microbiota^17^. However, the role of gut microbiota in the development of goose fatty liver is rarely investigated.

In this study, we hypothesized that goose as a superorganism has mechanisms promoting severe hepatic steatosis, but preventing the related harmful effects (e.g., inflammation), and that gut microbiota played a key role in these mechanisms. To test this, we performed an integrated analysis on the liver transcriptome and gut microbial metagenome in the overfed vs. normally fed geese. An *in vitro* study with goose primary hepatocytes was also conducted to provide some mechanistic insight. Our findings confirmed this hypothesis by identifying the gut microbiota and a suppressed complement system as prosteatotic and protective components in this unique NAFLD model.

## Results

### Severe Hepatic Steatosis was Induced in Overfed Geese

**Figure 1:**
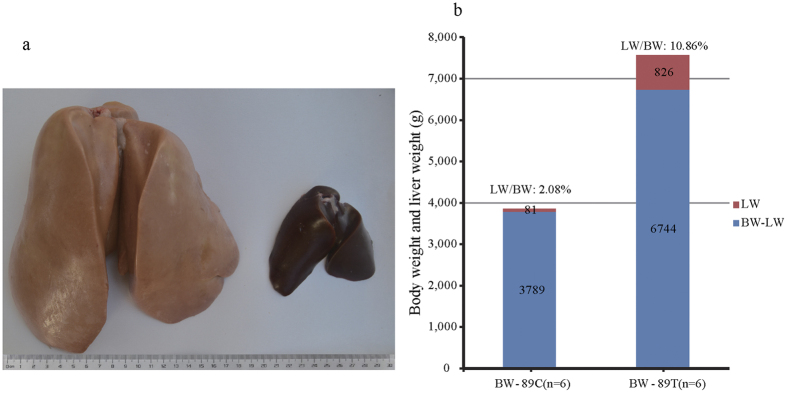
Body and liver weights were dramatically increased in the overfed vs. normally fed geese. (**a**) Representative images of fatty liver from an overfed Landes goose (treatment or T group, on the left) compared to normal liver from a normally fed Landes goose (control or C group, on the right) at 89 days of age (or at day 19 of overfeeding). (**b**) Body weight (BW), liver weight (LW) and the ratio of LW to BW in the overfed and normally-fed Landes geese at day 19 of overfeeding. N = 6. The data were presented as the means ± SD.

It is well known that a short period of overfeeding (2–3 weeks) can lead to dramatic increases in both body weight (BW) and liver weight (LW), as well as induce severe hepatic steatosis in geese^10^,^18^. Consistent with this, this study showed that, compared to control geese (3.87 ± 0.25 kg, n = 6), the BW of the overfed geese was greater (7.57 ± 0.48 kg) at day 19 of overfeeding. Correspondingly, the LW of the overfed geese (826.26 ± 182.26 g) was much greater than in the control geese (80.63 ± 12.80 g). Compared to the control, the ratio of LW to BW also increased in the overfed geese (10.86%) vs. the control geese (2.08%). In line with the drastic increase of liver weight in the overfed geese, the liver color turned from a normal liver color (deep red) to a fatty liver color (khaki). Blood biochemistry analysis indicated that the levels of triglyceride (TG), total cholesterol (CHO), high density lipoprotein cholesterol (HDL-C), glutamic-pyruvic transaminase (ALT), and glutamic oxalacetic transaminase (AST) were all significantly higher in the overfed geese than the control geese (*P* < 0.05), while the levels of low density lipoprotein cholesterol (LDL-C) and fasting blood glucose were not significantly different between the two groups of geese (Supplementary Table S1). The elevation of blood TG, CHO, HDL-C, ALT and AST is often associated with fatty liver in mammalian animals. Together, these findings suggested the overfeeding experiment was successful and severe steatosis was induced in the livers of the overfed geese (Fig. 1a,b).

### Liver Transcriptome Analysis

To illustrate how the goose liver transcriptome responded to overfeeding, cDNA libraries from the livers of the control and overfed geese at different times of overfeeding were constructed and sequenced. More than 90% of raw reads passed the filter with 25 to 37 million clean reads acquired from each library (Supplementary Table S2). The transcripts obtained from *de novo* assembly of the clean reads were matched to 13,032 and 13,536 unigenes, respectively, from chicken and duck genome databases, or a total of 13,973 annotated unigenes without redundancy (Supplementary Table S3). The length distributions of contigs, transcripts and unigenes are presented in Supplementary Fig. S1, and the detailed annotations of the unigenes are presented in Supplementary Table S4. All the unigenes with eggNOG IDs were clustered into proper categories (Supplementary Table S4). The distribution of the unigenes classified by gene function is presented in Supplementary Fig. S2.

**Figure 2:**
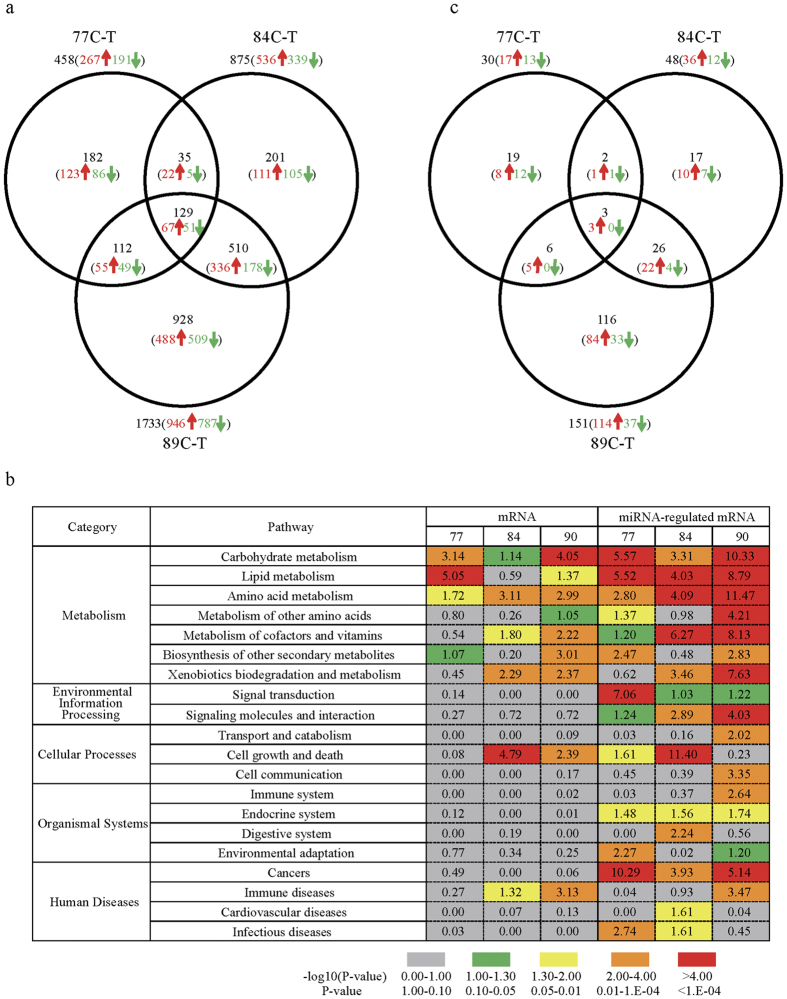
Outline of genes or microRNAs that differentially expressed in the goose livers. (**a**) The number of genes that were differentially expressed in the livers of the overfed (treatment or T group) vs. normally fed (control or C group) Landes geese at different time points of overfeeding (at 77-, 84- and 89 day of age or at day 7, 14 and 19 of overfeeding). (**b**) KEGG pathway analysis on the differentially expressed genes (DEGs) and microRNAs (DEMs)-targeted DEGs that were identified in the liver by the C-T comparisons at 77-, 84-, and 89 days of age. The DEM-targeted DEGs are predicted according to the procedures in the Methods section. The numbers presented on the right side of the table were transformed from *P*-values using the following formula: −log_10_ (*P*-value). The color bars at the bottom of the table denote the scale of the transformed *P*-values. (**c**) The number of microRNAs that were differentially expressed in the livers of the overfed vs. normally fed Landes geese at different time points of overfeeding. 77 C-T, 84 C-T and 89 C-T denote the C-T comparisons at 77-, 84- and 89 days of age, respectively. Numbers in black, red and green color represent the total, up-regulated and down-regulated numbers of DEG or DEM, respectively.

The DEGs in livers were identified between the overfed (T) and control groups (C) (Fig. 2a). A total of 458 (267 upregulated), 875 (536 upregulated) and 1,733 DEGS (946 upregulated) were identified at day 7, 14 and 19 of overfeeding, respectively, including 129 DEGs (67 upregulated) present throughout the study period (Supplementary Table S5). Five DEGs with the lowest *P*-values are shown in Table 1. Pathway analysis of the DEGs indicated that the carbohydrate, lipid and amino acid metabolism (in the KEGG category of metabolism) pathway was significantly enriched at day 7 of overfeeding (Fig. 2b). This transcriptome landscape subsequently shifted to that characterized by the metabolism pathway, the cell growth and death (in the category of cellular processes) pathway, and the immune diseases (in the category of human diseases) pathway at day 14 and 19 of overfeeding (Fig. 2b). Detailed information on the above is shown in Supplementary Table S6.

### Liver MicroRNA (miRNA) Analysis

As miRNAs regulate gene expression, we tested whether liver miRNAs responded to overfeeding in a way similar to the liver transcriptome. For this purpose, small RNA libraries constructed from 21 liver samples were deep sequenced (Supplementary Table S7). After data filtering, 15–30 bp-long reads were counted and grouped to generate unique reads. The length distributions of the total and unique sequences and the classification of the sequences are presented in Supplementary Fig. S3. The unique reads were annotated by aligning against miRNA databases available for 10 animal species. In total, 1,163 homologous miRNAs were identified. Their abundance distribution is shown in Supplementary Fig. S4. Ninety six miRNAs that were most highly expressed (>1,000 reads) accounted for 98.89% of 97,532,881 reads (Supplementary Table S8).

The differentially expressed miRNAs (DEMs) were identified between the C and T groups. A total of 30 (17 upregulated), 48 (36 upregulated) and 151 DEMs (114 upregulated) were identified at day 7, 14 and 19 of overfeeding, respectively (Fig. 2c, Supplementary Table S9). Five DEMs with the lowest *P*-values are shown in Table 2. From the aforementioned DEGs, the target genes of the DEMs were selected based on the notion that a miRNA generally down-regulates the expression of its target gene, and *vice versa* (Supplementary Table S10, Supplementary Fig. S5). Pathway analysis of the predicted target mRNAs indicated that the pathways enriched in the liver transcriptome almost overlapped those from miRNA analysis (Fig. 2b). This consistency illustrated miRNAs responded to overfeeding in a way similar to that of the liver transcriptome, and that miRNAs play an important role in shaping the liver transcriptome landscape.

### Intestinal Metagenome Analysis

As evidence indicates gut microbiota play a role in human NAFLD, we tested whether gut microbiota contributed to goose fatty liver by sequencing gut microbiomes from the experimental geese (Supplementary Table S11). In total, 3,628 out of 36,747 operational taxonomic units (OTUs) were identified with 97% sequence similarity. The OTU classification at the phylum level is shown in Supplementary Fig. S6 and Supplementary Table S12. On average, *Firmicutes* was the most abundant phylum to which 36.60% of sequences were assigned, followed by *Proteobacteria* (27.24%), *Bacteroidetes* (9.78%), *Cyanobacteria* (6.34%), *Actinobacteria* (6.06%), *Fusobacteria* (4.07%), *Spirochaetes* (2.62%), *Verrucomicrobia* (1.93%), *Acidobacteria* (1.21%), *Deferribacteres* (0.63%) and the other phyla (3.52%). *Firmicutes* more densely inhabited the duodena, jejuna and ilea than caeca in the overfed geese, and its abundance was influenced by overfeeding. In regard to *Bacteroidetes*, the third most abundant phylum, it had a relatively stable occupancy in duodena and jejuna, but appeared to be negatively affected in ilea and positively affected in caeca by overfeeding. *Actinobacteria, Verrucomicrobia* and *Acidobacteria* all accounted for certain proportions of microbiota in duodena, jejuna and ilea, and they appeared to be negatively affected by overfeeding in duodena. *Cyanobacteria* and *Deferribacteres* had the most remarkable changes as a result of overfeeding. Although sequences assigned to *Cyanobacteria* only made up 1–2% of sequences in the control geese, it composed a much higher proportion of sequences in duodena, jejuna and ilea of the overfed geese (i.e., 14.45–20.03%, 0.64–13%, and 2.27–9.45% at day 7, 14 and 19 of overfeeding, respectively). In contrast, 8.70% sequences were assigned to *Deferribacteres* in the caeca of the control geese, but less than 1% sequences were assigned to *Deferribacteres* in overfed geese. Moreover, the ratios of *Firmicutes/Bacteroidetes* varied with different intestinal segments, i.e., the ratios from the duodenum, jejunum and ileum were much larger than those from the caecum, especially in the overfed geese (Supplementary Table S13). Interestingly, the ratios from the duodenum, jejunum and ileum in the overfed geese were on average larger than those from the control geese, but the opposite was true with the caecum.

OTUs were also clustered at the genus level for a deeper insight into the changes in the composition of gut microbiota. In total, 739 taxonomic groups were identified (Supplementary Table S14). According to total OTU numbers, *Streptococcus, Lactobacillus, Oscillospira, Clostridium* and *Enterococcus* were the main genera from the *Firmicutes* phylum. *Enterobacteriaceae/g*__(genus unassigned), *Desulfovibrio, Gallibacterium, Zea* and *Bradyrhizobium* were the main genera from the *Proteobacteria* phylum. Detailed information of the main genera are shown in Supplementary Fig. S7.

**Figure 3:**
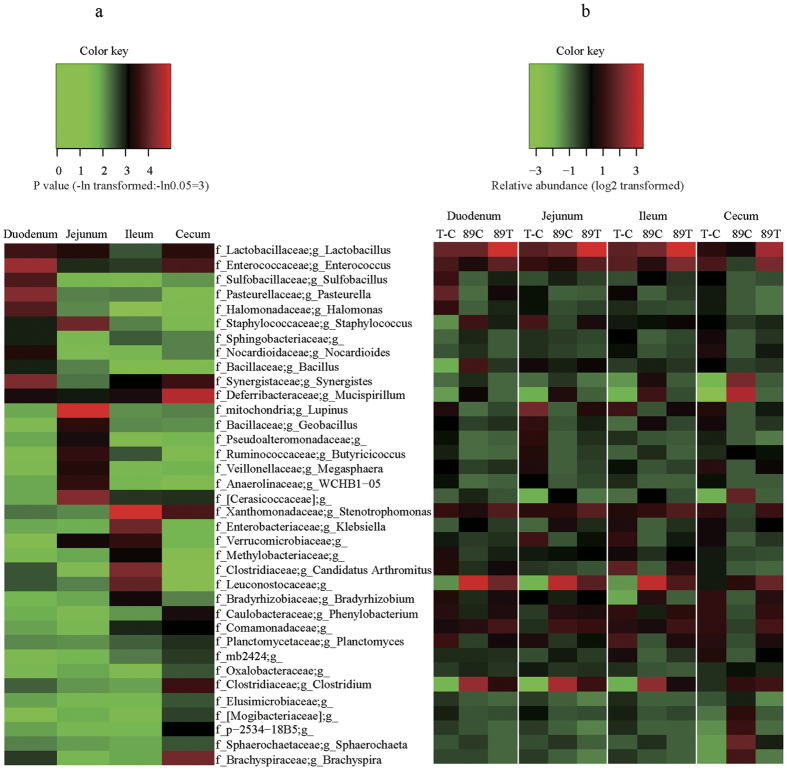
Heat maps of intestinal microbes that differentially inhabited different intestinal segments. (**a**) Heat map of transformed *P*-values, indicating the differences in the abundance of microbiomes that differentially inhabited the duodena, jejuna, ilea, and caeca of the overfed vs normally fed geese at day 19 of overfeeding. The transformed *P* values were calculated as –ln (*P* value). (**b**) Heat map of the log2 transformed abundance of microbes (indicated by 89C and 89T) in each intestinal segment of the overfed (treatment or T) and normally fed (control or C) geese, as well as the heat map of the log2 transformed difference in microbial abundance (indicated by T-C) between the two groups at 89 days of age. The normalization of the microbial abundance was conducted as follows: microbes of each genus that differentially inhabited the given intestinal segment were counted, followed by log2 transformation. The transformed abundance was then subtracted by the means of all the microbes in the intestinal segment, and the resultant difference was divided by the standard deviation of all log2 transformed abundance. The final values are the normalized microbial abundance for a certain genus of microbes. The color key represents the scale of the given variable in each map. In the names of microbes, ‘f’ and ‘g’ denote family and genus, respectively. The missing name after ‘g’ denotes that the genus was unassigned.

After normalization, the relative abundance of a variety of genera and species were calculated and compared between the C and T groups at day 19 of overfeeding (Fig. 3 and Supplementary Table S15). In total, 36 genera were significantly (*P* < 0.05) affected by overfeeding in at least one intestinal segment. For instance, 5 genera, *Lactobacillus, Enterococcus, Sulfobacillus, Pasteurella* and *Halomonas*, significantly increased in abundance in the duodena of the overfed versus control geese, while 6 genera, *Staphylococcus, Sphingobacteriaceae/g*__(genus unassigned), *Nocardioides, Bacillus, Synergistes* and *Mucispirillum*, decreased in abundance. Remarkably, the abundance of *Lactobacillus* and *Enterococcus* increased in all intestinal segments of the overfed versus control geese, whereas the abundance of *Mucispirillum* decreased. Moreover, the abundance of some bacteria varied with different intestinal segments. Typically, *Geobacillus* more densely inhabited the jejunum than other intestinal segments; in contrast, *Bacillus, Bradyrhizobium* and *Clostridium* less densely inhabited the duodenum, ileum, and cecum, respectively.

### Involvement of Gut Microbiota in Liver Response to Overfeeding

**Figure 4:**
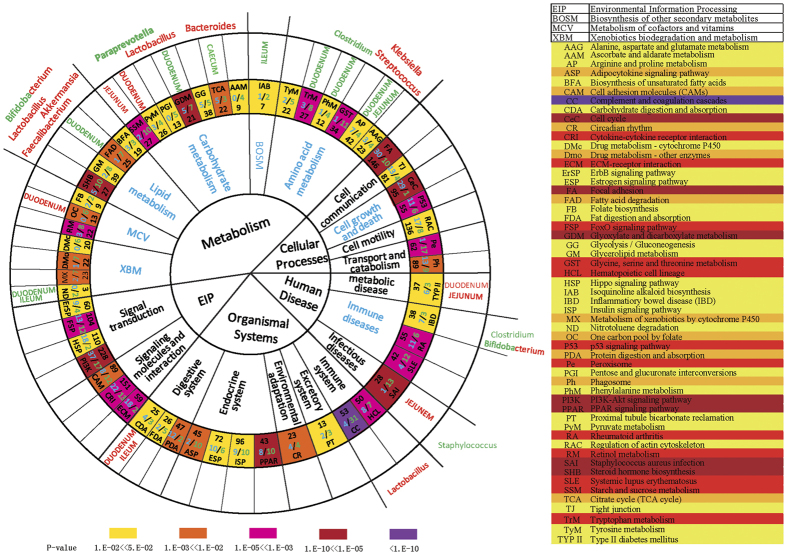
Intestinal microbes responded in synergy with the response of goose liver transcriptome to overfeeding. The enriched KEGG pathways were revealed by the genes (DEGs) differentially expressed in the livers or the genes of microbes that differentially inhabited intestines of the overfed vs. normally fed geese at day 19 of overfeeding. The circles from the center outward denote the first-, second-, and third-tier KEGG pathways, respectively. The second-tier pathways in blue are statically significantly enriched. The abbreviated names of the pathways, the total numbers (in black) of genes in each pathway, and the numbers (in blue) of the upregulated/downregulated genes in the livers of the overfed vs normally-fed geese are denoted in the circle with the third-tier pathways. The background colors in this circle indicate the *P*-values for a given pathway (with color bar for the scale of *P*-values at the bottom). The circle with different intestinal segments indicates the genes of gut microbes shared the same pathways with the DEGs from liver transcriptome analysis. The names of intestinal segments in red or green denote the abundance of microbes were increased or decreased in the given intestinal segments, respectively. In the circle with microbial names, the microbes whose abundances were increased or decreased in the overfed vs normally-fed geese are denoted in red or green, respectively. The microbes in mixed color are those whose abundance was increased or decreased over different intestinal segments. The table on the right illustrates the abbreviated KEGG pathways.

To determine whether gut microbiota contributed to the liver transcriptome, we performed pathway analyses on the genes of gut microbes that were dramatically influenced by overfeeding. Firstly, the analysis indicated that the gut microbiota could synergize the response of goose liver to overfeeding through a number of signaling pathways (Fig. 4, Supplementary Table S16). For instance, the ‘metabolism’ pathways featured in the liver transcriptome overlapped with those revealed by the genes of gut microbes that were prominently influenced by overfeeding, particularly those involved in fatty acid degradation, citrate cycle, and arginine and proline metabolism. In addition, three other pathways were also significantly influenced by overfeeding in both liver transcriptomes and metagenomes, including those involved in type II diabetes mellitus, *staphylococcus aureus* infection, and carbohydrate digestion and absorption. Secondly, the synergetic effect of gut microbes varied with different intestinal segments. For example, the microbes in the duodenum appeared to be more closely related to ‘metabolism’ pathways than those in other intestinal segments as the microbes in the duodenum shared more signaling pathways with the liver transcriptome than those in other intestinal segments. Thirdly, a third-tier KEGG pathway analysis identified some gut microbes with known functions that could contribute to the changes in the liver transcriptome landscape (Fig. 4). For instance, the pathway related to fatty acid degradation was suppressed in the livers of the overfed geese at day 19 of overfeeding (7 downregulated vs 1 upregulated genes), and this pathway was also closely related to the microbes in the duodenum, including *Lactobacillus, Akkermansia, Faecalibacterium* and *Bifidobacterium*, which are known to be involved in lipid metabolism^19^,^20^,^21^.

As generation of short chain fatty acids (SCFA) is a function of microorganism, we determined the levels of SCFA in the plasma of the overfed vs normally-fed geese at day 19 of overfeeding (Supplementary Table S17). Data showed that, compared to the normally-fed geese, butyric acid but no other SCFA (i.e., acetic acid, propionic acid and isobutanoic acid) in plasma was significantly (*P* < 0.05) increased in the overfed geese. The increase in plasma butyric acid could be due to the increased amount of *Butyricicoccus* in the gut as previous study indicates that *Butyricicoccus pullicaecorum* is a butyrate producer^22^. Indeed, *Butyricicoccus* increased in abundance in the jejuna of the overfed geese (Supplementary Table S15). As butyrate can decrease TNF production and inhibit inflammatory responses through NFκB inhibition in the pathogenesis of Crohn’s disease^23^, the increased plasma butyric acid in the overfed geese may contribute to the protective mechanism against inflammation in goose fatty liver. Together, gut microbiota played a synergistic role in liver response to overfeeding by generation of their products, and activation or suppression of the same signaling pathways.

### Suppression of Complement Components by Microbial Metabolites

**Figure 5:**
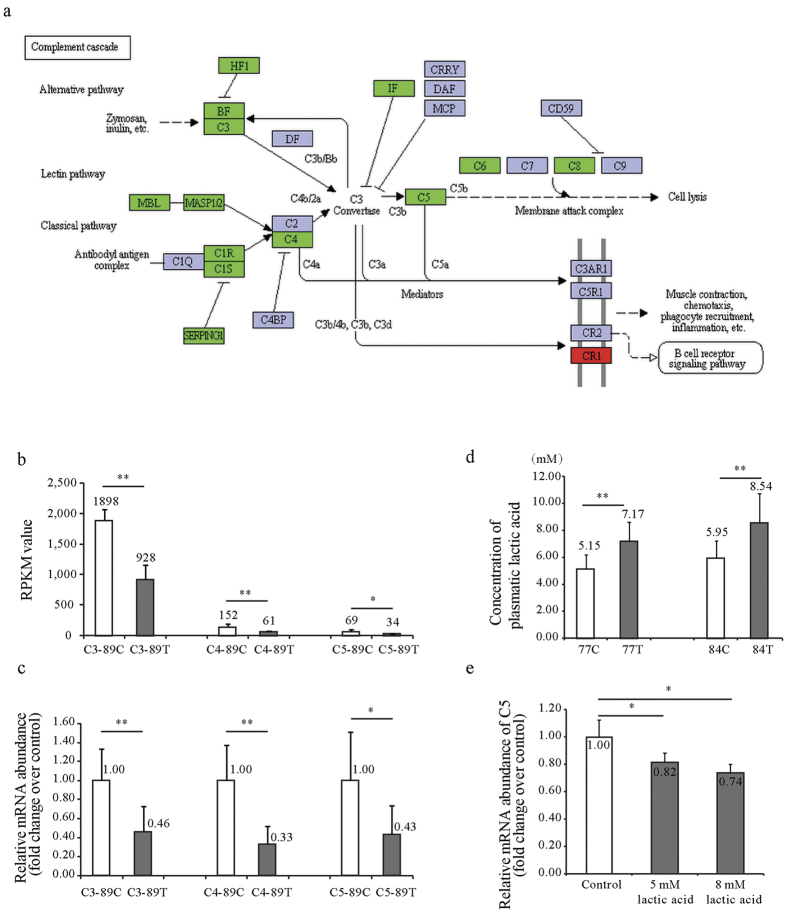
The suppression of the ‘Complement cascades’ pathway by lactic acid from intestinal *Lactobacillus*. (**a**) The genes in the ‘Complement system’ KEGG pathway were differentially regulated in the livers of the overfed vs normally fed geese. The genes in red/green stand for those whose expressions were upregulated or downregulated by overfeeding. (**b**) The comparisons of *C3*, *C4* and *C5* expression in the livers of the overfed (the treatment or T group) and normally-fed geese (the control or C group) at day 19 of overfeeding. The data was from liver transcriptome analysis. N = 3. (**c**) The expression of *C3*, *C4* and *C5* genes in the livers of the overfed vs normally-fed geese at day 19 of overfeeding. The data was from quantitative real time PCR analysis. N = 6. (**d**) The concentrations of plasma lactic acid in the overfed vs normally fed geese at day 7 and 14 of overfeeding. N = 6. (**e**) The expression of *C5* was determined by quantitative PCR in the goose primary hepatocytes treated with vs without lactic acid. N = 5. All the data are presented as the means ± SD.

To identify the mechanism by which gut microbiota contributed to the liver transcriptome in response to overfeeding, we further investigated an exemplary pathway: the suppressed ‘Complement and coagulation cascades’ pathway (Fig. 5a). We firstly performed quantitative PCR to validate the expression of three key complement component genes (*C3*, *C4* and *C5*) in the livers at day 19 of overfeeding. Consistent with transcriptome analyses, they were all suppressed in goose fatty liver (Fig. 5b,c). As *Lactobacillus* can protect against NAFLD by attenuating inflammation in mice^24^,^25^ and was most significantly influenced by overfeeding, we hypothesized that *Lactobacillus* could regulate the complement system through its metabolite, lactic acid. In line with the increased abundance of *Lactobacillus* in the intestines of the overfed versus normally fed geese, the plasma level of lactic acid was also significantly increased in the overfed geese at both day 7 and 14 of overfeeding (Fig. 5d). Importantly, the expression of *C5* was significantly (P < 0.05) suppressed in goose primary hepatocytes treated with 5 mM and 8 mM lactic acid versus control cells (Fig. 5e). These findings provide direct evidence for the contribution of gut microbiota to the goose liver transcriptome in response to overfeeding.

### Suppression of *TNFα* by Lactic Acid via *HNF1α/C5* Pathway

**Figure 6:**
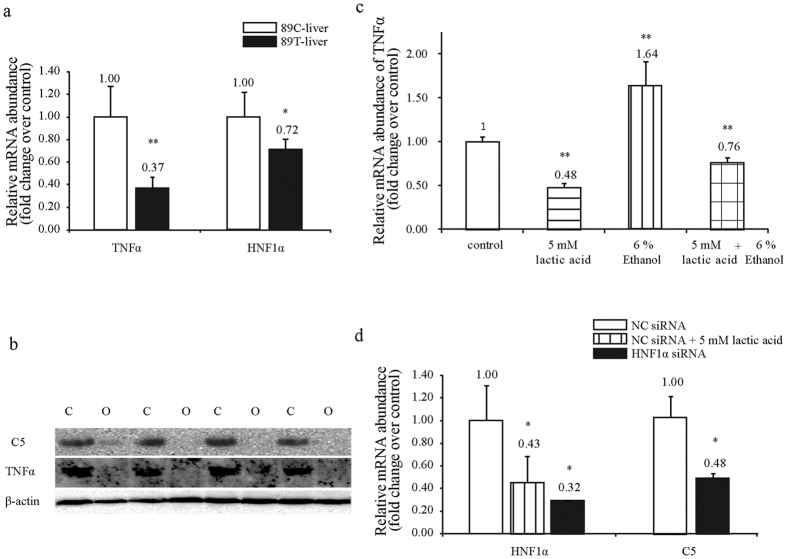
The inhibition of *TNFα* in goose hepatocytes by lactic acid through *HNF1α/C5* pathway. (**a**) The expression of *TNFα* and *HNF1α* at mRNA level was suppressed in the livers of the overfed vs. normally fed geese. N = 6. (**b**) The expression of *C5* and *TNFα* at protein level was suppressed in the livers of the overfed vs. normally fed geese. N = 4. (**c**) Ethanol induced *TNFα* expression was inhibited by lactic acid in goose primary hepatocytes. N = 4. (**d**) *C5* expression was inhibited in goose primary hepatocytes transfected with siRNA targeting to *HNF1α* vs. negative control (NC) siRNA N = 4. *^,^**denote P < 0.05 and P < 0.05, respectively. Data in (**a,**c) and (**d**) are presented as the means ± SD.

To shed light on the mechanism by which inflammatory and fibrotic responses were inhibited in goose fatty liver, we conducted a series of *in vitro* studies with cultured goose primary hepatocytes. The expression of *C5* and *TNFα* (a potent proinflammatory and immunoregulatory cytokine) in the liver was first determined by quantitative PCR and Western immunoblot analyses. Data showed that the suppression of *C5* at both mRNA and protein levels was associated with the suppression of *TNFα* in goose fatty liver vs. normal liver (Fig. 6a,b). To determine whether lactic acid could inhibit the expression of *TNFα*, the cultured goose primary hepatocytes were treated with lactic acid, ethanol or both. The hepatocytes without these treatments were used as control. Data showed that lactic acid could suppress the ethanol induction of *TNFα* (Fig. 6c), supporting that *C5* mediates the suppression of *TNFα* or inflammation by lactic acid in goose fatty liver.

To understand how *C5* was suppressed by lactic acid, we cloned and sequenced the upstream sequence of goose *C5* (accession number: KX171333), followed by prediction of *HNF1α* as a potential transcription factor of goose *C5* (Supplementary Table S18). *HNF1α* was also predicted as a transcription factor using the upstream sequence of human *C5*. The expression of *HNF1α* was suppressed in goose fatty liver vs. normal liver (Fig. 6a). Consistently, lactic acid treatment inhibited the expression of *HNF1α* in the cultured cells (Fig. 6d), suggesting lactic acid could downregulate the expression of *C5* by inhibiting the expression *HNF1α*.

We treated goose primary hepatocytes with a control negative siRNA (NC siRNA) or siRNA targeted to *HNF1α* (*HNF1α* siRNA). Quantitative PCR analysis indicated that *HNF1α* siRNA successfully reduced the expression of *HNF1α* by about 70% compared to NC siRNA treatment (Fig. 6d). The reduction of *HNF1α* led to a significant decrease in the expression of *C5* (Fig. 6d). The result confirmed that *HNF1α* mediated the suppression of *C5* by lactic acid. Taken together, the findings from this *in vitro* study indicated that HNF1α/*C5* mediated lactic acid-induced suppression of TNFα in goose fatty liver.

## Discussion

NAFLD presents a great challenge to public health around the world. Goose is a unique animal model with the discovery of a hepatic protective mechanism. In this study, we gained insight into the mechanism underlying overfeeding-induced goose fatty liver by integrating liver transcriptome and gut microbial metagenome analyses.

From the transcriptome analysis, 458 DEGs identified at day 7 of overfeeding shaped the liver transcriptome landscape, which was characterized by the ‘metabolism of carbohydrate, lipid and amino acid’ pathways. The DEGs included upregulated (e.g., *Gpi, Pdh, Cs, Acly, Acc, Fasn, Scd* and *Dgat2,* the key enzymes involved in fatty acid synthesis) and downregulated DEGs (e.g., *Apob*^26^ and *Lpl*^27^, the key genes involved in lipid packing and release) which were in accordance with previously published results^15^. The upregulated and downregulation DEGs cooperatively promoted the formation of fatty liver at early stages of overfeeding. Additionally, some genes (e.g., *Cs, Scd*, *Dgat2, Apob,* and *Lpl*) retained the same expression pattern throughout the overfeeding period. During later stages of overfeeding, the ‘cell growth and death’ and the ‘immune diseases’ pathways jointly shaped the liver transcriptome landscape. When *de novo* synthesis of lipids surpassed the capacity of initial hepatocytes (i.e., day 14 and 19 of overfeeding), hepatocyte enlargement or proliferation occurred to allow for more fat storage, which involved the ‘cell growth and death’ pathway (e.g., *Tgfb3*, *Cdk1* and *Ccna2* with known functions in cell growth and proliferation, and *P53*, *Tnfsf10* and *Prkar2b* genes in regulation of cell death, see Supplementary Table S6 for other relevant DEGs). Consistent with this, miRNA profiling analysis also revealed that similar pathways were involved. However, the number of DEMs was far fewer than those of DEGs, suggesting some DEMs had multiple target genes. Thus, these DEMs played a key role in the development of goose fatty liver.

When hepatocytes were overloaded with lipids, lipotoxicity occurred. Lipotoxicity has been linked to the increase of oxidative stress, endoplasmic reticulum stress, inflammation, cell death, and human diseases^28^. Surprisingly, immune disease-related pathways (systemic lupus erythematosus and rheumatoid arthritis) appeared to be suppressed in goose fatty liver, as relevant DEGs were downregulated rather than upregulated. In line with this, the third-tier KEGG pathway analysis indicated that the complement system, a key player in the immune system and inflammation, was also inhibited in goose fatty liver, which may explain the suppression of immune diseases. These findings were consistent with our recent data showing the induction of adiponectin receptors (which cleave proinflammatory ceramides^29^) and the suppression of *TNFα* (a potent proinflammatory and immunoregulatory cytokine^29^,^30^) by overfeeding in goose fatty liver, which is the opposite in mammalian NAFLD^31^,^32^. We thus proposed that hepatic inflammation was inhibited in goose fatty liver despite severe steatosis.

The complement system consists of a cascade of proteases and soluble factors, including complement components, receptors and regulatory factors^33^. As a key system for immune surveillance and homeostasis^34^, it is also involved in other processes, such as development, regeneration and metabolic diseases^35^. As key components, both *C3* and *C5* are central to the activation of its three pathways: the classical pathway, the lectin pathway, and the alternative pathway (Fig. 5a). Recent studies indicate that the complement system is activated in human/rodent livers with AFLD^36^ or NAFLD^37^. Moreover, C3 deletion inhibits hepatic steatosis induced by alcohol in mice^38^. Deletions of *C5*^36^ and *C1q*^39^ also alleviate hepatic steatosis and suppress the expression of inflammatory factors (TNFα and IL6) induced by alcohol. In this study, the ‘complement and coagulation cascades’ pathway was comprehensively suppressed in goose fatty liver (Fig. 5a), especially at day 19 of overfeeding. This suppression was validated by quantitative PCR analysis of *C3*, *C4* and *C5* (Fig. 5c) and Western blot analysis of *C5*, which, together with the suppressed ‘immune diseases’ pathway, suggests that these suppressed pathways may be key components of the protective mechanism preventing progression from steatosis to steatohepatitis in goose liver.

In regard to gut microbiota, data indicated that *Firmicutes* (36.60%), *Proteobacteria* (27.24%), and *Bacteroidetes* (9.78%) were the most abundant phyla in goose gut microbiota, and the abundance of *Firmicutes* was influenced by overfeeding and varied with different intestinal fragments. Although the increase of *Proteobacteria* is associated with the progression of NAFLD (inflammation and fibrosis) in human and mouse probably through production of endogenous ethanol^40^, it seemed that *Proteobacteria* was not an important player in the development of goose fatty liver as the average abundance of *Proteobacteria* were barely different between the control and overfeeding groups of geese (Supplemental Table 12). This is consistent with the notion that goose fatty liver is less injured from severe steatosis. *Firmicutes* and *Bacteroidetes* can both produce SCFA through fermentation using high fat diets (for *Firmicutes*) or high plant fibers (for *Bacteroidetes*)^41^. *Firmicutes* was likely the primary producer of SCFAs promoting goose fatty liver, especially at later stages of overfeeding, as its abundance was increased. Moreover, recent studies showed that the ratio of *Firmicutes/Bacteroidetes* was associated with mammalian obesity, where NAFLD may occur. For example, Schwiertz *et al*. reported a decrease of this ratio in the gut of obese people^42^, while Turnbaugh *et al*. observed an increase of this ratio in the gut of obese mice and obese humans^43^. Consistent with the latter study, the ratios from the duodena, jejuna and ilea of overfed geese were on average greater than those in control geese. However, the ratio from the caeca was smaller, suggesting the ratio varied with intestinal segments. In regard to *Cyanobacteria*, the microbes more densely inhabited the duodena, jejuna and ilea of overfed geese than control geese. *Cyanobacteria* may promote goose fatty liver via its products, cyanotoxins, as it is known that some microbial toxins, like tunicamycin, can induce fatty liver. In addition, gut microbiota may contribute to the regulation of ‘lipid metabolism’, ‘amino acid metabolism’, and ‘immune diseases’ pathways in goose fatty liver, as some pathways revealed by enriched microbial genes were shared by liver DEGs.

In this study, we also noticed that *Lactobacillus, Akkermansia* and *Bifidobacterium* increased in abundance, but *Clostridium* decreased in the overfed versus control geese. It is known that *Lactobacillus* is involved in carbohydrate metabolism^44^, lipid metabolism^19^ and the immune system^24^. *Lactobacillus* also plays a protective role in the development of fatty liver^24^,^25^,^45^. Consistent with these findings, this study showed that *Lactobacillus* was indeed involved in the same pathways. We also demonstrated the increase of *Lactobacillus* contributed to suppression of the complement system in goose fatty liver via its metabolite, lactic acid, as blood lactic acid was significantly higher in the overfed geese than the control geese and that *C5* was suppressed in the goose primary hepatocytes treated with 5 mM and 8 mM lactic acid compared to a control. Moreover, *Akkermansia* is associated with inflammation in fat tissues of patients with type 2 diabetes^46^, thus we speculated that the increase of *Akkermansia* contributed to the suppression of the immune system pathway in goose fatty liver. Lastly, the decrease of *Clostridium* could reduce the risk of alcohol-induced steatohepatitis as it has been shown that *Clostridium* can produce alcohol in an animal’s intestine^47^. Altogether, these findings suggest gut microbiota are a component of the protective mechanism preventing a progression from steatosis to steatohepatitis in goose liver.

In conclusion, goose, like many other animals, is a complex system in which its symbiotic microbes coordinately respond to external stimuli, such as overfeeding. When geese are overfed, the liver transcriptome landscape gradually shifted from being characterized by the metabolism pathways to one characterized by more pathways including the ‘cell growth and death’ pathway and the ‘immune diseases’ pathway, in addition to the ‘metabolism’ pathways. Gut microbiota (predominantly *Firmicutes*) contributed to shaping the landscape via the gut-liver axis. The enriched phyla/genera varied in different intestinal segments, which is usually neglected in human studies due to limitations on sample collection. Remarkably, the complement system was suppressed in goose fatty liver, at least partially, due to increased blood lactic acid produced by the enriched *Lactobacillus*. This study thus provides an insight into goose liver capacity for and tolerance to severe hepatic steatosis.

## Methods

### Ethics Statement

All animal protocols were approved by the Institutional Animal Care and Use Committee (IACUC) of the Yangzhou University Animal Experiments Ethics Committee with permission number SYXK(Su) IACUC 2012–0029, and carried out in accordance with the approved guidelines. All efforts were made to minimize the suffering of the animals. The movement of birds was not restricted before the age of 60 days, but all the birds were restricted as they were moved to cages for the period of adaptation and overfeeding. The experimental geese were killed with an electrolethaler before harvesting their liver samples and intestinal contents. For isolation of goose primary hepatocytes, the embryos at 23 days of hatch were decapitated with a pair of scissors before harvesting their livers.

### Animals, Experiment Design and Sampling

A total of 42 healthy 65-day-old Landes geese were purchased from Rui Nong Farm (Yangzhou, China) and randomly divided into a control group and an overfeeding group (or treatment group) (n = 24 and 18, respectively). The geese were raised with a homemade feed before 65 days old (Supplementary Table S19). A 5-day-long pre-overfeeding was performed to prepare the overfeeding group of geese for formal overfeeding, which lasted 19 days. The overfeeding procedure and diet regimes were performed as previously described^48^. The routine husbandry management was carried out through the experiments. During the time of overfeeding, the geese from control group were sacrificed at 70, 77, 84 and 89 days old of age (6 geese each time point) for sample collection (samples were named as 70C, 77C, 84C, and 89C, correspondingly), while the geese from the overfeeding group were sacrificed at 77, 84 and 89 days old of age (6 geese each time point) for sample collection (samples were named as 77T, 84T, and 89T, correspondingly). Blood, liver and intestinal content samples were harvested from the geese. Blood samples were used to determine the levels of lactic acid, SCFA, and other blood variables. Liver samples were snap frozen in liquid nitrogen and stored at −70 °C for later use. The intestinal content samples from duodenum, jejunum, ileum and cecum were also collected (see sampling position in Supplementary Fig. S8), and the samples were transferred at 4 °C and stored at −20 °C. For transcriptome and metagenome analyses, samples from 3 geese per group were used with liver samples at each time point, including 70C, 77C, 77T, 84C, 84T, 89C and 89T, and the intestinal content samples from duodenum, jejunum, ileum and cecum, including 77T, 84T, and 89T for the overfed geese and 89C for the control geese. The analyses were performed by Shanghai Personal Biotechnology Co., Ltd.

### Transcriptome Sequencing

The mRNA sequencing was performed as previously described with minor modifications^49^. Briefly, RNA samples were prepared using Illumina TrueSeq RNA Sample Preparation Kit (Illumina Inc., San Diego, USA). The concentrations of liver RNA samples from different individuals were adjusted to 10 nM. Sequencing of the cDNA was performed by Personalbio (Shanghai, China) using the Illumina Hiseq system.

For mRNA analysis, the raw reads obtained from sequencing were filtered using Fast QC software. The clean reads were then used for the *de novo* assembly of transcripts by Trinity software^50^. The obtained transcripts were mapped to the chicken (*Gallus gallus*, Galgal4 version) and duck (*Anser platypus*, BGI_duck_1.0 version) genomes retrieved from the following website: https://www.ensembl.org using NCBI Blast (NCBI Blast + v2.2.26), respectively. The mapping to the duck genome was a supplement to that to the chicken genome. The mapped reads of each gene were calculated and presented as Reads Per Kb per Million reads (RPKM)^51^. The significance was determined using DESeq (http://bioconductor.org/packages/release/bioc/html/DESeq.html). Genes with max RPKM value >2, fold change of treatment over control >2 or < 0.5, and *P*-value < 0.05 were assigned as differential expressed genes (DEGs). Kyoto Encyclopedia of Genes and Genomes (KEGG) Automatic Annotation Server (KAAS, http://www.genome.jp/tools/kaas/) was used to perform signaling pathway analysis.

### Small RNA Sequencing

The small RNA sequencing and miRNA analysis was performed as previously described with minor modifications^49^. Briefly, sequencing of the small RNA libraries was performed by Personalbio using the Illumina Miseq system. The clean reads with 15–30 bp (obtained by trimming adapter sequences and removing the low-quality sequences from raw reads) were counted and grouped for identical sequences. The resultant unique reads were then BLAST against the non-coding RNA (ncRNA) database Rfam (11.0) to assess the quality of sequences and to annotate the obtained ncRNAs. The reads were also searched against the miRNA databases (miRBase, v20.0) of 10 known species (i.e., *Bombyx mori/Bmo, Bos Taurus/Bta, Drosophila pseudoobscur/Dps, Gallus gallus/Gga, Homo sapiens/Has, Mus musculus/Mmu, Ornithorhynchus anatinus/Oan, Rattus norvegicus/Rno, Taeniopygia guttata/Tgu, and Zea mays/Zm*) to identify goose miRNA homologs. The reads of miRNAs were calculated and presented as reads per million reads (RPM, which equals to the ratio of the count of miRNA to total count of clean reads multiplied by 1,000,000). To determine differential expressed miRNAs (DEMs), only the miRNAs with at least two RPM values >1 were used. The *P*-values were calculated between the overfeeding group and the control group using a two-tail t-test. The miRNAs with both fold change of the overfeeding group over the control group >1.5 or < 2/3 and *P*-value < 0.05 were considered DEMs. Target genes of the identified DEMs were predicted using miRanda with the TargetScan principle^52^ and further refined using RNAhybird software by constraint of minimum free energy value (MFE < −30 kcal/mol)^53^. All predicted target DEGs were from the liver transcriptome analysis abovementioned. The final pairs of DEMs to DEGs were chosen according to the following rule: DEMs were upregulated while their target DEGs were downregulated by overfeeding, and *vice versa*. KEGG Automatic Annotation Server (KAAS, http://www.genome.jp/tools/kaas/) was used to perform signaling pathway analysis.

### Metagenome Sequencing

The Metagenome sequencing and analysis was performed as previously described with minor modifications^54^. Briefly, DNA was extracted from intestinal contents samples using QIAamp DNA stool mini kit (QIAGEN, Cat. No. 51504). The variable regions (V4) of the 16S ribosomal DNA were amplified by PCR for individual sample.

The primers (forward primers: 5′AYTGGGYDTAAAGNG3′, reverse primers: 5′TACNVGGGTATCTAATCC 3′) and conditions for PCR and amplicon purification were performed according to the protocols as previously described^55^. Using the pair-end method, barcoded V4 amplicons were sequenced by Illumina Miseq with a 6 cycle index read. The sequences with an average Phred score lower than 25, ambiguous bases, homopolymer run exceeding 6, mismatches at the sites of primers, or lengths shorter than 100 bp were all removed. Only the sequences with an overlap longer than 10 bp and perfect match were assembled. The reads that were unable to be assembled were discarded. The barcodes and the sequencing primers were trimmed from assembled sequences. The trimmed sequences were then uploaded to MGRAST for further analysis.

To gain the abundance of each taxon for gut microbes, the trimmed sequences were compared to the RDP database with the classification option ‘best hit’. The cutoffs of e-value, sequence identity, and the minimum alignment length were set at 8, 97%, and 120 bp, respectively. The abundance of gut microbes from each sample was calculated at family level, which was done by MGRAST. The abundance at genus level was transformed by log2 and normalized as previously described^55^, which made the global mean value equal to zero and the standard deviation equal to one. The t-test was performed to determine the statistical significance. Functional prediction was performed using Phylogenetic Investigation of Communities by Reconstruction of Unobserved States (PICRUSt) software^56^, and KEGG Automatic Annotation Server (KAAS, http://www.genome.jp/tools/kaas/) was used to perform signaling pathway analysis.

### Determination of Plasma Lactic Acid and SCFA

Plasma samples obtained from the overfed geese and normally fed geese at day 7 and 14 of overfeeding were used for determination of plasma lactic acid, 6 each group). The concentration of lactic acid was measured with a Lactic Acid (LD) Detection Kit (Cat. No. A019-2, Nanjing Jiancheng Bioengineering Institute, China) according to the manufacture’s instruction. Plasma samples obtained from the overfed geese and normally fed geese at day 19 of overfeeding were used for SCFA determination 6 each group). The method for determination of plasma SCFA concentration was described in Supplementary Methods.

### Acquisition of Goose *C5* Upstream Sequence and Prediction of Transcription Factor

The upstream sequence of goose *C5* was amplified and sequenced with primers that were designed according to the upstream sequence of duck *C5* (forward: 5′CTTCCCGTAAGGGCTGAC3′, reverse: 5′GGATTCAACTCTATCTGGCT3′). The 2000 bp upstream sequence of human *C5* was retrieved from Ensembl database. The prediction of transcription factor was performed with the ‘Match’ program (www.gene-regulation.com) using the default parameters.

### Preparation of Goose Primary Hepatocytes

Hepatocytes were isolated from Landes goose embryos at 23 days of hatch. The preparation was performed as previously described^48^. After the primary hepatocytes were obtained, the cells were diluted with culture medium to 1 × 10^6^ cells/mL, plate 1 × 10^6^ cells per well in 12-well dishes, followed by incubation in 5% CO_2_ incubator at 38 °C until treatment. The media was renewed at first 6 h of incubation.

### Treatment of Goose Primary Hepatocytes with Lactic Acid and Ethanol

For lactic acid treatment, the cultured goose primary hepatocytes were treated with complete culture media containing 0, 5 and 8 mM lactic acid (Cat. No. 69785-250 ML, Sigma, USA) for 5 h. For lactic acid/ethanol treatment, goose primary hepatocytes were treated with 5 mM lactic alone, 6% ethanol alone (Sigma, USA), or 5 mM lactice plus 6% ethanol for 12 h, respectively. The cells treated with complete culture media were used as control.

### Assay with HNF1α Interfering RNA in Goose Primary Hepatocytes

HNF1α siRNA (sense: 5′GCAACCACCAUGGGAACAATT3′, antisense: 5′UUGUUCCCAUGGUGGUUGCTT3′) synthesized by GenePharma Co., Ltd, Shanghai, China) and negative control siRNA (sense: 5′ UUCUCCGAACGUGUCAUCUTT3′, antisense: 5′ACGUGACACGUUCGGAGAATT3′) were used to transfect goose primary hepatocytes using X-tremeGene siRNA Transfection Reagent (Cat. No. 04476093001, Roche, Germany) according to the manufacture’s instruction. The amount of 40 pmol each siRNA were used for each well of cells in 12-well dish. After 36 hr of transfection with the siRNAs,. the cells were harvested with TRIzol Reagent (Cat. No. 15596026, Life, USA).

### RNA Isolation, cDNA Synthesis, and Quantitative Real-time PCR (qRT-PCR)

Total RNA were extracted from liver samples or primary hepatocytes and cDNA was synthesized with the purified RNA samples as previously described^57^. The expression of the complement components, including C3, C4, *C5* and TNFα, was determined by qRT-PCR as previously described^58^. Goose glyceraldehyde-3-phosphate dehydrogenase gene (*GAPDH*) gene was used as an internal control. The primer sequences of the genes were listed in Supplementary Table S20. The expression levels of C3, C4, and *C5* genes were normalized to the control gene. The cycle threshold (Ct) was determined with the supplied software. The message RNA abundance of genes of interest was calculated using 2^−ΔΔCt ^59 and presented as fold change over control.

### Protein Assay and Immunoblot Analysis

Protein concentration of each liver sample was determined as previously described^60^. The protocol for immunoblot analysis was also previously described^60^. The following antibodies were used in this study: Complement *C5* (Cat. No. bs-15197R, Beijing Biosynthesis Biotechnology Co., Ltd. Beijing, China), *TNFα* (Cat. No. bs-10802R, Beijing Biosynthesis Biotechnology Co., Ltd. Beijing, China) and *β-Actin* (Cat. No. sc-47778; Santa Cruz Biotechnology, Inc., Santa Cruz, CA).

### Statistical Analysis

The data were expressed as the means ± SD. SPSS 16.0 (SPSS China, Shanghai, China) was used to perform the Student’s *t*-test or one-way analysis of variance for statistical significance of differences between or among different treatments. A *P* < 0.05 was considered statistically significant.

## Additional Information

**How to cite this article**: Liu, L. *et al*. Prosteatotic and Protective Components in a Unique Model of Fatty Liver: Gut Microbiota and Suppressed Complement System. *Sci. Rep.* **6**, 31763; doi: 10.1038/srep31763 (2016).

## Supplementary Material

Supplementary Information

Supplementary Table S1

Supplementary Table S2

Supplementary Table S3

Supplementary Table S4

Supplementary Table S5

Supplementary Table S6

Supplementary Table S7

Supplementary Table S8

Supplementary Table S9

Supplementary Table S10

Supplementary Table S11

## Figures and Tables

**Table 1:** The upregulated or downregulated genes with 5 lowest *P* values in the goose livers^a^.

77 C-T			84 C-T			89 C-T		
Gene Symbol	P-value	Fold change^b^	Gene Symbol	P-value	Fold change	Gene Symbol	P-value	Fold change
up-regulated genes								
* Thrsp*	1.8E-26	5.6	*Bkj*	1.3E-68	39	*Wdr93*	1.6E-56	62
* Wdr93*	4.5E-26	13	*Npas2*	9.8E-52	20	*Anks4b*	1.8E-41	70
* Inhbb*	4.4E-23	5.5	*Ptgr1*	3.5E-33	6.5	*Mfap2*	4.5E-38	63
* G0s2*	2.0E-20	12	*C9orf172*	1.3E-22	12	*Ccdc80*	9.2E-33	16
* *PLIN2	4.7E-20	9.0	*Myom1*	8.2E-21	8.3	*Map3k7cl*	2.6E-29	45
down-regulated genes								
* Cmpk2*	7.9E-17	0.18	*Tinag*	9.0E-44	0.09	*Mt4*	5.7E-95	0.01
* Bmf*	2.3E-14	0.24	*Cyp1a1*	4.6E-40	0.12	*Cyp1a1*	3.7E-79	0.01
* Itih4*	2.1E-13	0.16	*Epas1*	2.9E-32	0.16	*Igfbp2*	2.3E-57	0.03
* Cdk9*	1.1E-11	0.36	*Adh6*	1.9E-31	0.16	*Mc5r*	3.9E-56	0.03
* Fpgs*	4.8E-11	0.37	*Igfbp4*	3.7E-24	0.21	*Slco1b3*	6.7E-40	0.05

^a^The livers were harvested from the overfed (T) vs normally-fed (C) geese at 77-, 84-, and 89 days of age.

^b^The fold change is calculated by dividing the expression level of the differentially genes in the livers of the overfed geese by that of the normally fed geese.

**Table 2:** The upregulated or downregulated microRNAs with 5 lowest *P* values in the goose livers^a^.

77 C-T			84 C-T			89 C-T		
miRNA	P-value	Fold change^b^	miRNA	P-value	Fold change	miRNA	P-value	Fold change
up-regulated miRNA								
oha-let-7g-3p	1.3E-03	9.5	oha-miR-129a-5p	2.0E-04	3.3	efu-miR-223	4.0E-05	3.1
ssa-miR-23b-3p	3.3E-03	2.4	oha-miR-33a-5p	3.5E-03	1.9	csa-let-7d	5.1E-05	2.1
oha-miR-129b-3p	4.5E-03	2.3	efu-miR-7b	4.5E-03	1.7	cgr-miR-221-5p	1.7E-04	15
oha-miR-23b-3p	5.5E-03	1.9	oha-miR-126-3p	4.9E-03	3.1	efu-miR-221	2.0E-04	7.6
oha-miR-182-5p	5.5E-03	1.6	oha-miR-27b-5p	5.3E-03	2.4	oha-let-7e-5p	2.2E-04	1.7
down-regulated miRNA								
hsa-miR-6087	9.7E-03	0.46	chi-miR-454-3p	1.4E-03	0.63	oha-miR-29a-3p	2.3E-04	0.39
chi-miR-301a-5p	1.0E-02	0.30	oha-miR-30a-5p	1.5E-03	0.63	tch-miR-15a-5p	3.1E-04	0.52
gga-miR-7475-5p	1.1E-02	0.54	sme-miR-10b-5p	2.9E-03	0.56	ipu-miR-1388	4.3E-04	0.50
cgr-miR-1260	1.7E-02	0.58	oha-miR-30b-5p	3.1E-03	0.51	efu-miR-26c	7.7E-04	0.61
mmu-miR-5100	1.8E-02	0.60	oha-miR-10b-5p	3.7E-03	0.50	tch-miR-27a-3p	8.8E-04	0.66

^a^The livers were harvested from the overfed (T) vs normally-fed (C) geese at 77-, 84-, and 89 days of age.

^b^The fold change is calculated by dividing the expression level of the differentially genes in the livers of the overfed geese by that of the normally fed geese.
