# Supplementary material for: Prosteatotic and Protective Components in a Unique Model of Fatty Liver: Gut Microbiota and Suppressed Complement System
Source: Sci Rep. 2016 Aug 23;6:31763. doi: 10.1038/srep31763 (PMC4994046; doi:10.1038/srep31763)
Supplement: Supplementary Information [file srep31763-s1.pdf]

Supplementary Materials for

Prosteatotic and Protective Components in a Unique Model of Fatty Liver: Gut Microbiota and Suppressed Complement System

Long Liu[1], Xing Zhao[1], Qian Wang[1], Xiaoxian Sun[1], Lili Xia[1], Qianqian Wang[1], Biao Yang[1], Yihui Zhang[1], Sean Montgomery[2], He Meng[3], Tuoyu Geng[1*], and Daoqing Gong[1*]

Correspondence should be addressed to T. G. (tygeng@yzu.edu.cn) or D.G. (yzgong@163.com).

This file includes:

Supplementary Figure 1 to 8 (pp 2-7)

Supplementary Table 1 to 17 (pp 8-14)

Supplementary Methods (pp 15)

Supplementary Figures

Supplementary Figure 1. The length distributions of contigs, transcripts and unigenes that were identified by analysis of goose liver transcriptome

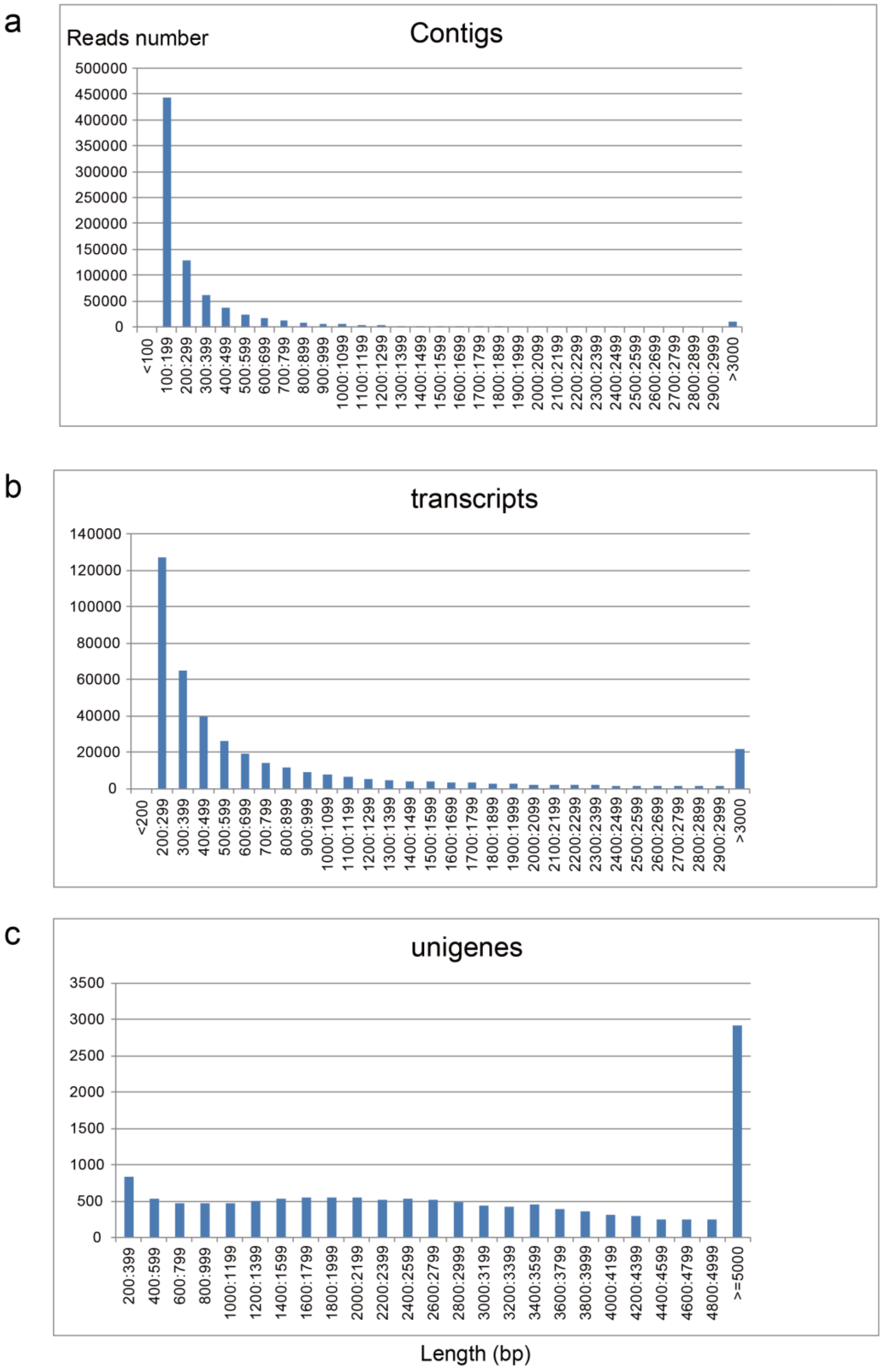

Supplementary Figure 2. The distribution of the unigenes classified by gene function

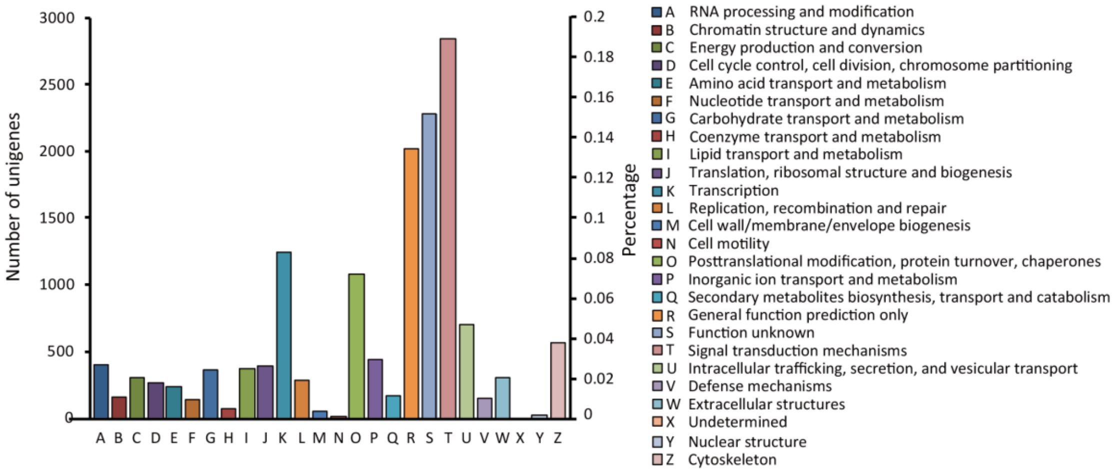

The abbreviation illustration was present on the right.

Supplementary Figure 3. The distribution of the sequences obtained from small RNA sequencing

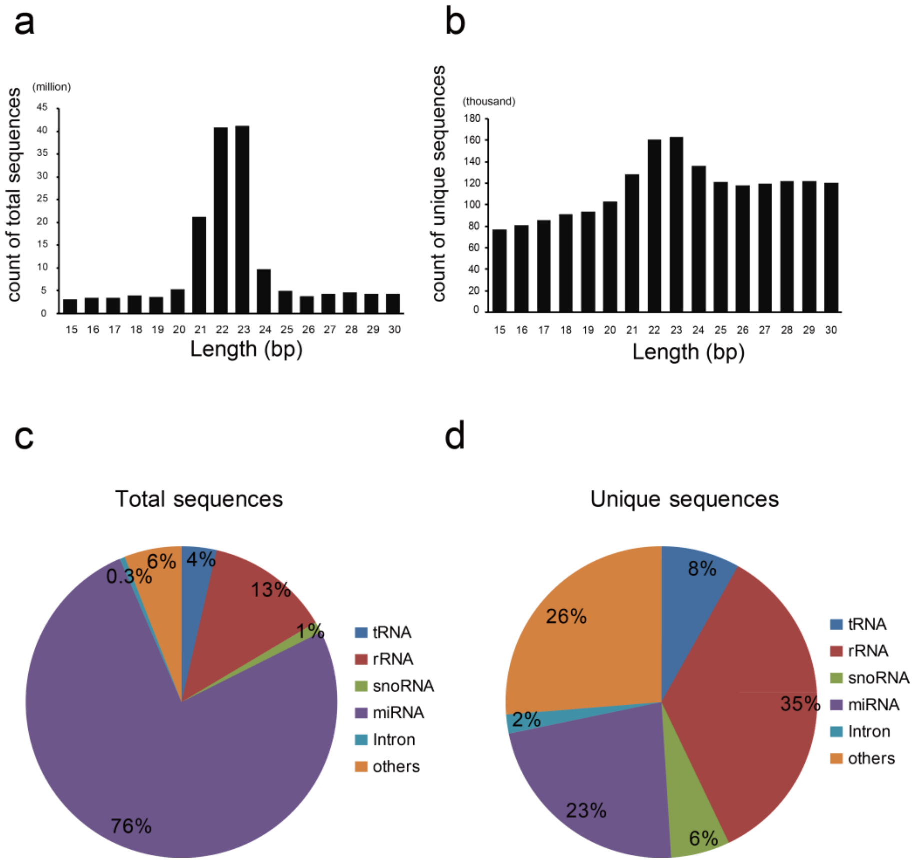

(a) Length distribution of the sequences obtained from small RNA sequencing. (b) The abundance distribution of unique sequences with 15-30 bp long selected from small RNA sequences. (c) The abundance of different types of small RNAs obtained from small RNA sequencing. (d) The abundance of unique small RNAs by type obtained from small RNA sequencing.

Supplementary Figure 4. The abundance distribution of miRNAs categorized by the reads number

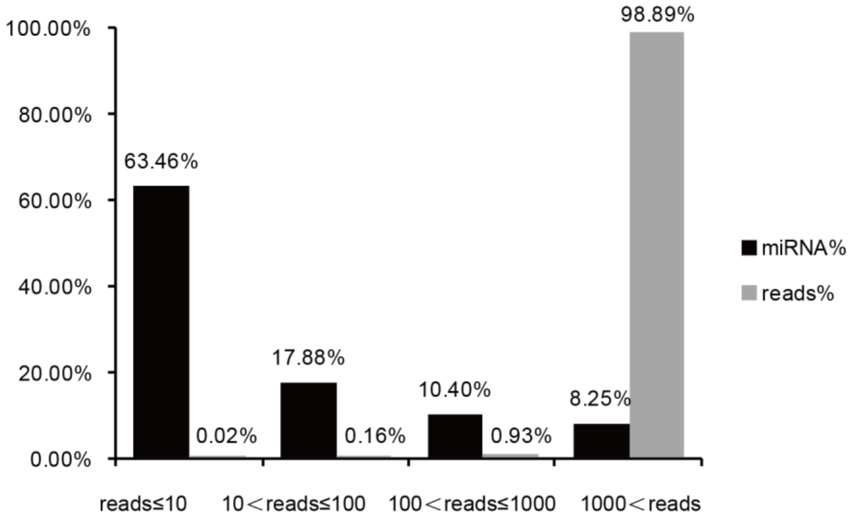

Supplementary Figure 5. Predicted target genes of differentially expressed miRNAs (DEMs) using the differentially expressed genes (DEGs) from liver transcriptome analysis as a source

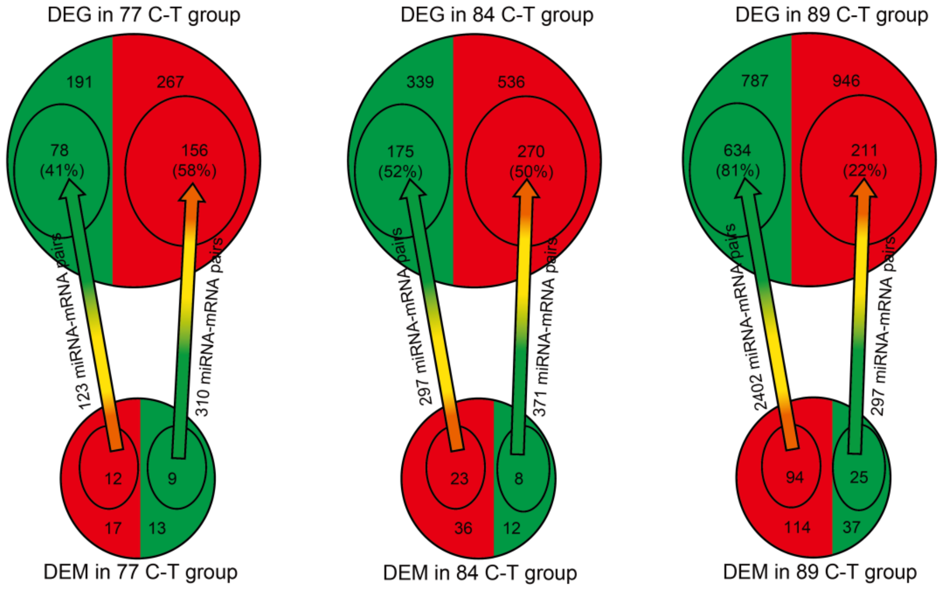

77C-T, 84 C-T and 89 C-T denote the comparisons between the control and overfed groups at day 7, 14 and 19 of overfeeding, respectively. Red or green background colors indicate DEM and DEG upregulated or downregulated in overfed group versus control group, respectively.

Supplementary Figure 6. **The OTU classifications at phylum level with microbial abundance indicated using data from intestinal metagenome analysis**

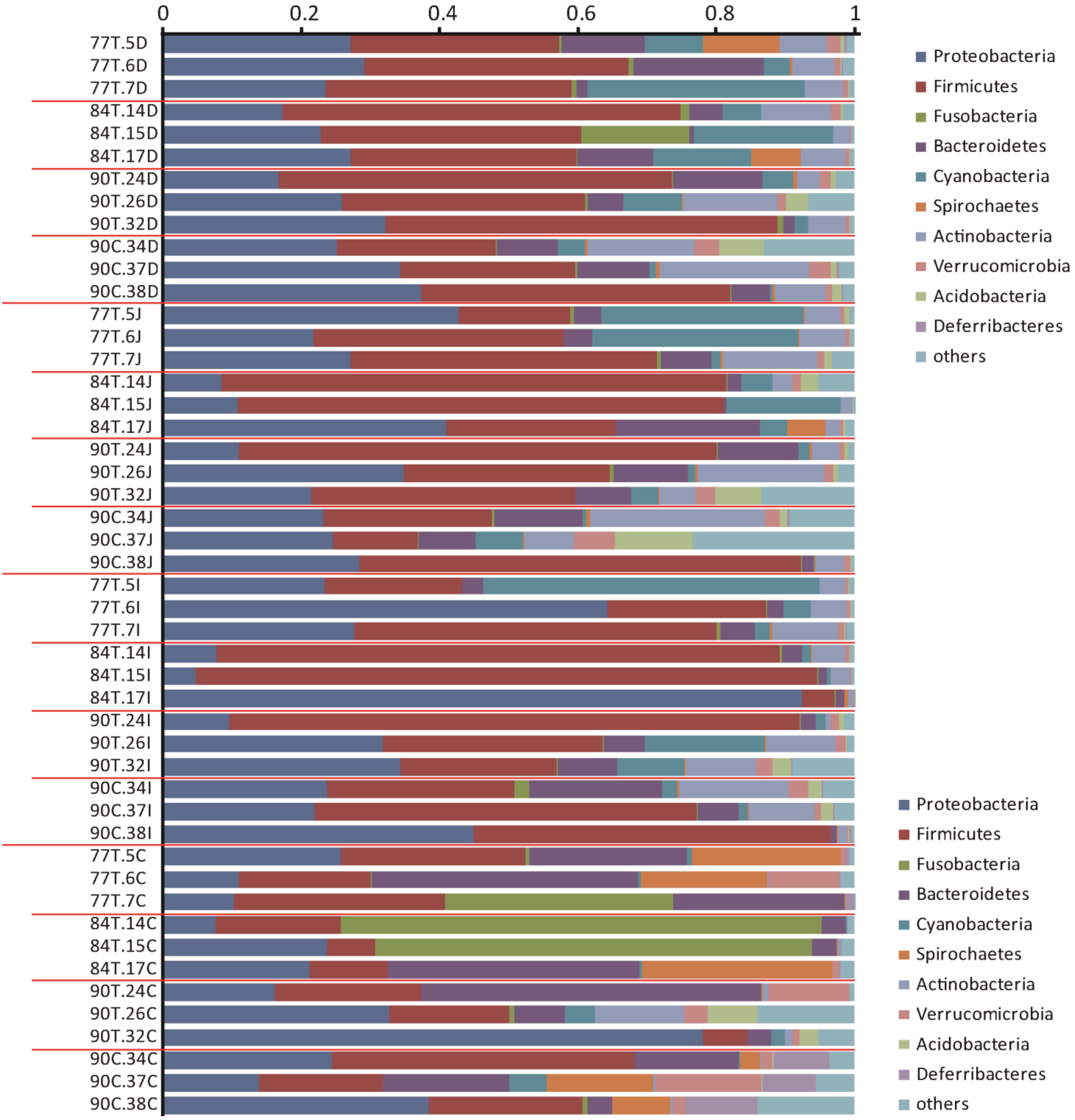

In the sample identities shown on the left, 77T, 84T and 89T denote that the microbes are from the intestinal segments (i.e., D for duodenum, J for jejunum, I for ileum, and C for caecum) of the overfed geese at 77, 84, and 89 days of age, respectively. 77C, 74C and 89C are correspondingly for the control geese 7,14 and 19 of overfeeding, 89C denotes control group at day 19 of overfeeding and. The color bars are illustrated on the right. N=3 for each group.

Supplementary Figure 7. The abundance distribution of the main genera in ten most abundant phyla revealed by metagenomics analysis of goose intestinal microbiota

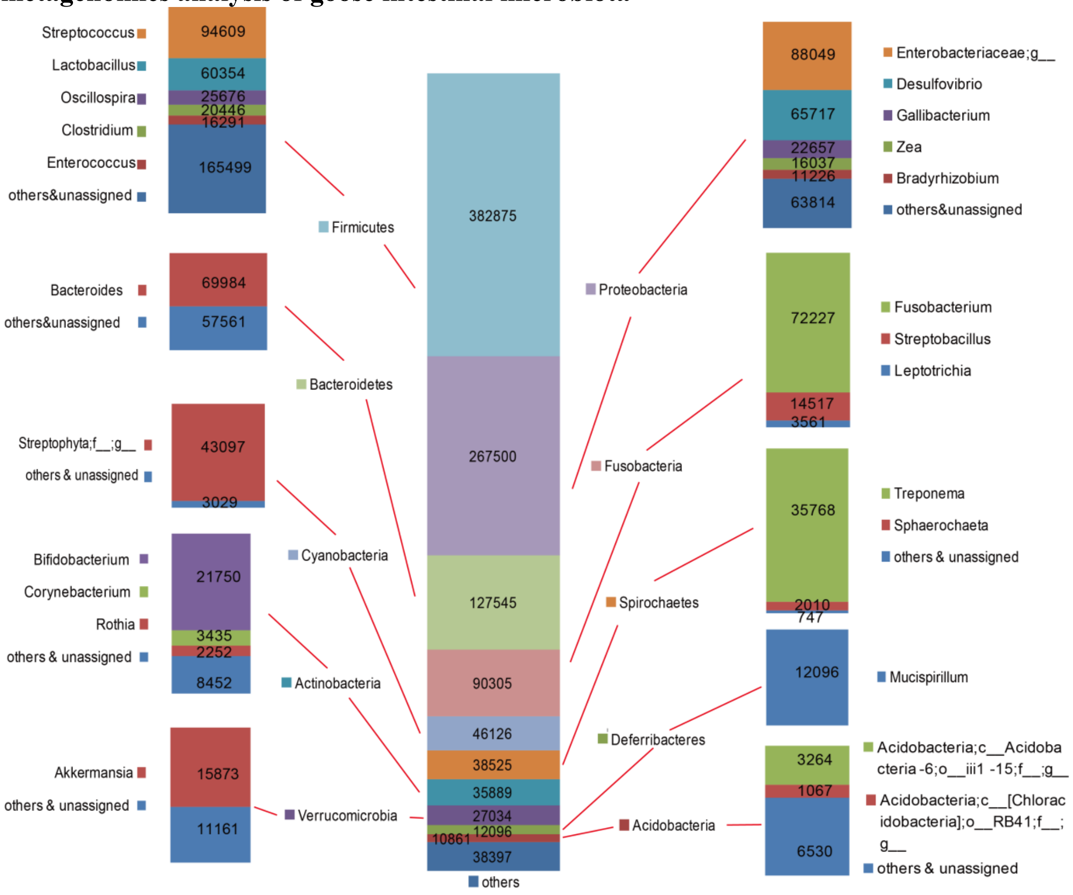

The color bars in the center of the figure indicate the ten most abundant phyla while the color bars on the left and right sides of the figure indicate the main genera of the corresponding phyla. In the names of microbes, ‘c’, ‘o’, ‘f’ and ‘g’ denote class, order, family and genus, respectively. The missing names after ‘c’, ‘o’, ‘f’ and ‘g’ denote that the class, order, family or genus were unassigned.

Supplementary Figure 8. The image illustrate the sites for the intestinal content samples collected from different segments including duodenum, jejunum, ileum and caecum, which are framed by rectangles

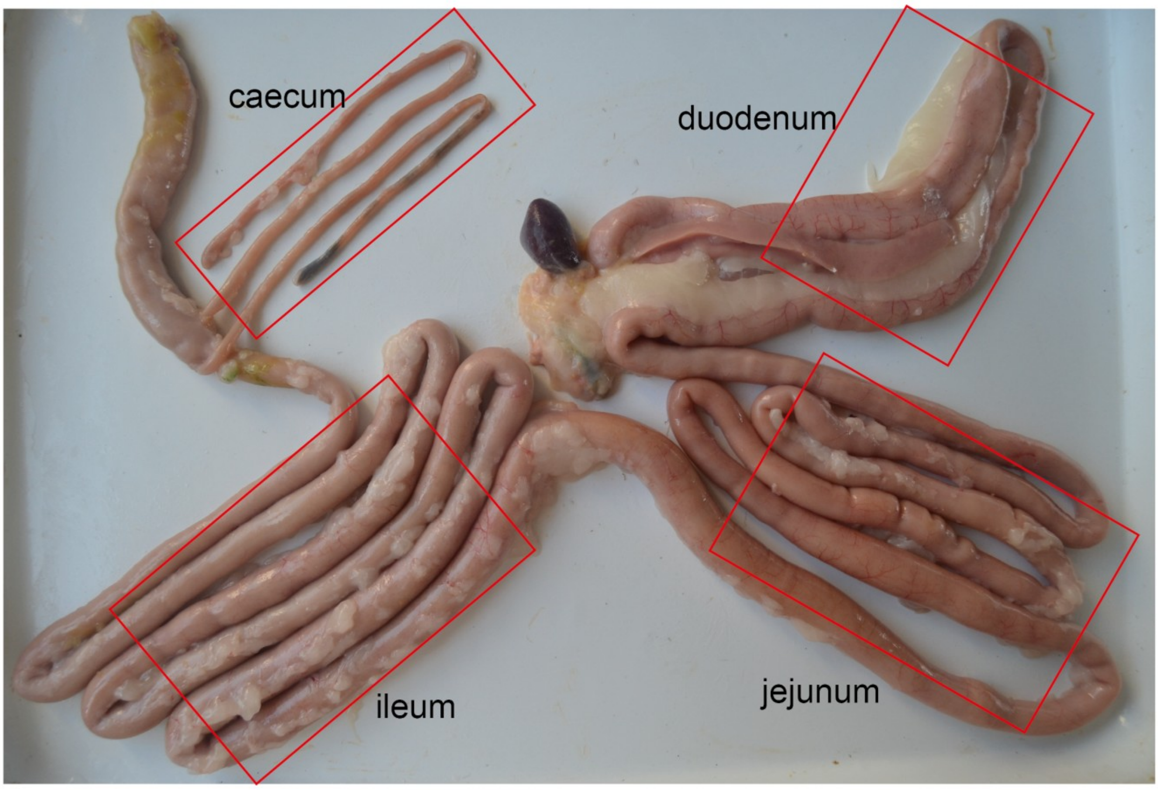

Supplementary Tables

Supplementary Table S1. Blood chemistry in the overfed Landes geese vs the control geese at day 14 of overfeeding

	Number	Control	Overfeeding
ALT/ ($U.L^{-1}$)	6	$8.25^{A}\pm1.30$	$63.75^{B}\pm11.86$
AST/ ($U.L^{-1}$)	6	$8.25^{A}\pm1.30$	$210.00^{B}\pm53.39$
CHO/($nmol.L^{-1}$)	6	$3.49^{A}\pm0.38$	$5.17^{B}\pm1.13$
TG/($nmol.L^{-1}$)	6	$0.84^{A}\pm0.75$	$2.32^{B}\pm0.88$
HDL-C/($nmol.L^{-1}$)	6	$2.24^{A}\pm0.20$	$3.45^{B}\pm0.99$
LDL-C/($nmol.L^{-1}$)	6	0.94±0.21	0.78±0.12
Glucose/($mmol.L^{-1}$)	6	10.18±0.29	10.67±0.17

Different superscripts denote $P<0.05$. ALT, glutamic-pyruvic transaminase; AST, glutamic oxalacetic transaminase; CHO, total cholesterol; TG, triglyceride; HDL-C, high-density lipoprotein-cholesterol; LDL-C, low-density lipoprotein-cholesterol. Glucose levels were measured using a glucometer (Sinocare; ChangSha, Inc.). Other parameters were determined using Hitachi 7170 Chemistry Analyzer.

Supplementary Table S2. An outline of the raw data and clean data from transcriptome sequencing

Sample[a]	Reads	Raw Reads	Raw Data(bp)	Q20[b] (%)	GC(%)	Clean Reads	Clean Reads (%)	Clean Data(bp)	Clean data[c] (%)
70C-1	Paired	37,728,211	9,432,052,750	96.37	56.13	34,674,153	91.91	8,465,168,372	89.75
70C-3	Paired	41,642,993	10,410,748,250	96.18	56.12	37,889,558	90.99	9,268,556,024	89.03
70C-4	Paired	40,867,433	10,216,858,250	96.35	55.41	37,441,691	91.62	9,172,367,994	89.78
77T-5	Paired	36,062,072	9,015,518,000	96.28	57.21	32,992,330	91.49	8,085,501,361	89.68
77T-6	Paired	35,101,185	8,775,296,250	97.58	57.51	33,077,203	94.23	8,157,125,073	92.96
77T-7	Paired	32,973,964	8,243,491,000	97.11	55.98	30,621,860	92.87	7,511,107,612	91.12
77C-10	Paired	27,545,454	6,886,363,500	97.12	55.84	25,592,671	92.91	6,262,019,368	90.93
77C-12	Paired	34,956,997	8,739,249,250	97.06	56.76	32,398,904	92.68	7,939,511,716	90.85
77C-13	Paired	29,154,022	7,288,505,500	97.05	56.17	26,984,319	92.56	6,631,363,801	90.98
84T-15	Paired	31,992,710	7,998,177,500	97.13	57.91	29,725,149	92.91	7,273,695,913	90.94
84T-16	Paired	30,891,977	7,722,994,250	97.32	57.47	28,868,937	93.45	7,134,946,787	92.39
84T-17	Paired	28,791,293	7,197,823,250	97.42	57.04	26,972,184	93.68	6,666,367,906	92.62
84C-20	Paired	28,846,543	7,211,635,750	97.57	56.23	27,193,301	94.27	6,720,187,661	93.19
84C-22	Paired	27,899,427	6,974,856,750	97.36	56.53	26,179,554	93.84	6,460,986,485	92.63
84C-23	Paired	27,088,691	6,772,172,750	97.25	56.49	25,309,133	93.43	6,251,042,980	92.30
89T-26	Paired	28,831,029	7,207,757,250	97.14	57.87	26,906,752	93.33	6,637,490,460	92.09
89T-31	Paired	32,104,319	8,026,079,750	97.26	56.52	30,064,264	93.65	7,420,448,058	92.45
89T-32	Paired	39,713,938	9,928,484,500	97.30	57.07	37,258,203	93.82	9,196,140,475	92.62
89C-36	Paired	29,286,979	7,321,744,750	97.45	56.16	27,567,173	94.13	6,808,635,682	92.99
89C-37	Paired	36,016,380	9,004,095,000	97.37	56.71	33,775,020	93.78	8,340,797,228	92.63
89C-38	Paired	33,064,526	8,266,131,500	97.28	56.36	30,932,766	93.55	7,634,181,817	92.35

[a] 70C-1, 3 and 4 are the liver samples from the normally fed geese (control or C group) at 70 days of age; 77T-5, 6 and 7 are the liver samples from the overfed geese (treatment or T group) at 77 days of age (or at day 7 of overfeeding); the liver samples harvested at 84 and 89 days of age are similarly named. N=3 for each group.

[b] denotes the percentage of data with base calling accuracy higher than 99%.

[c] denotes the proportion of clean data in raw data.

Supplementary Table S3. Summary of contigs, transcripts and unigenes from liver transcriptome analysis

	Total length	Number	Max length	Average length	GC (%)
Contig	303,720,385	802,368	27,786	378.53	47.07
transcript	334,838,335	391,049	27,786	856	48.08
unigene	46,600,489	13,973	27,786	3,335	49.26

Supplementary Table S4. The detailed annotations of unigenes from liver transcriptome analysis

See a separate supplementary file: Supplementary Table S4.xlsx

Supplementary Table S5. Detailed information on differentially expressed genes in the livers of the overfed vs normally fed geese

See a separate supplementary file: Supplementary Table S5.xlsx

Supplementary Table S6. Detailed information on differentially expressed genes in the 'carbohydrate metabolism, lipid metabolism, amino acid metabolism, cell growth and death and immune diseases' pathways

See a separate supplementary file: Supplementary Table S6.xlsx

Supplementary Table S7. An outline of raw data and clean data from small RNA sequencing

Sample[a]	No. of Raw Reads	No. of Clean Reads	Clean Reads (%)	No. of Reads between 15-30 nt	No. of Reads mapped to Rfam[a]	Reads mapped to Rfam (%)
70C-1	15,031,800	10,438,660	69.44	7,581,396	6,014,608	79.33
70C-3	11,986,407	8,194,500	68.36	5,984,715	4,967,801	83.01
70C-4	11,092,808	7,704,667	69.46	5,914,644	5,011,662	84.73
77T-5	10,726,369	7,582,036	70.69	5,788,205	4,943,948	85.41
77T-6	8,964,612	6,362,103	70.97	4,893,824	3,955,075	80.82
77T-7	11,452,336	7,859,957	68.63	5,101,201	4,296,818	84.23
77C-10	10,079,181	7,229,215	71.72	5,763,364	4,597,991	79.78
77C-12	11,808,753	8,480,073	71.81	7,135,624	6,258,326	87.71
77C-13	17,539,435	14,743,019	84.06	11,645,613	9,360,350	80.38
84T-15	13,755,617	12,015,071	87.35	9,527,134	8,271,424	86.82
84T-16	13,344,954	11,589,900	86.85	9,106,981	7,639,201	83.88
84T-17	13,608,421	11,488,307	84.42	8,931,022	7,782,528	87.14
84C-20	10,674,166	9,520,982	89.20	8,193,572	7,256,559	88.56
84C-22	12,252,717	10,456,564	85.34	8,652,289	7,331,380	84.73
84C-23	12,047,368	10,378,200	86.14	8,470,924	7,448,008	87.92
89T-26	10,592,451	9,146,103	86.35	6,747,546	5,712,806	84.66
89T-31	14,088,213	12,183,064	86.48	9,426,969	8,232,975	87.33
89T-32	13,109,131	10,695,514	81.59	7,001,663	5,750,326	82.13
89C-36	11,925,590	10,616,818	89.03	9,296,907	8,615,462	92.67
89C-37	11,766,552	10,248,238	87.10	7,922,801	6,868,075	86.69
89C-38	10,979,608	9,567,437	87.14	7,487,180	6,283,083	83.92

[a] 70C-1, 3 and 4 are the liver samples from the normally fed geese (control or C group) at 70 days of age; 77T-5, 6 and 7 are the liver samples from the overfed geese (treatment or T group) at 77 days of age (or at day 7 of overfeeding); the liver samples harvested at 84 and 89 days of age are similarly named.N=3 for each group.

[b]denotes the Rfam database which is a collection of RNA families.

Supplementary Table S8. List of most highly expressed miRNAs in the liver

See a separate supplementary file: Supplementary Table S8.xlsx

Supplementary Table S9. Detailed information on the differentially expressed microRNA in the livers of the overfed (treatment or T group) vs. normally fed geese (control or C group)

See a separate supplementary file: Supplementary Table S9.xlsx

Supplementary Table S10. Predicted target genes of differentially expressed miRNAs using differentially expressed genes identified in liver transcriptome analysis as a source of target genes

See a separate supplementary file: Supplementary Table S10.xlsx

Supplementary Table S11. An outline of the sequences obtained from gut microbial metagenome analysis

Group	Individual	Intestinal segment	No. of valid sequence	No. of High quality sequence	Group	Individual	Intestinal segment	No. of valid sequence	No. of High quality sequence
77T	77T-5	Duodenum	23,976	7,152	89T	89T-24	Duodenum	49,790	30,305
		Ileum	29,534	19,464			Ileum	37,361	29,636
		Jejunum	20,014	6,602			Jejunum	63,643	45,338
		caecum	23,087	17,999			caecum	86,810	63,126
	77T-6	Duodenum	23,869	9,267		89T-26	Duodenum	18,238	6,749
		Ileum	17,012	8,627			Ileum	28,663	18,026
		Jejunum	19,938	10,783			Jejunum	25,226	7,772
		caecum	49,390	36,922			caecum	36,480	20,354
	77T-7	Duodenum	25,310	12,153		89T-32	Duodenum	57,802	44,637
		Ileum	22,740	4,491			Ileum	37,622	11,583
		Jejunum	20,530	4,551			Jejunum	56,795	34,277
		caecum	35,218	25,057			caecum	63,803	50,050
84T	84T-14	Duodenum	58,606	30,639	89C	89C-34	Duodenum	21,958	7,578
		Ileum	66,333	33,015			Ileum	28,957	5,608
		Jejunum	78,089	55,282			Jejunum	33,922	4,299
		caecum	137,774	93,263			caecum	56,565	38,164
	84T-15	Duodenum	25,963	16,747		89C-37	Duodenum	47,647	8,546
		Ileum	19,534	8,826			Ileum	31,496	7,476
		Jejunum	21,949	14,390			Jejunum	51,358	30,707
		caecum	36,959	23,273			caecum	48,532	39,144
	84T-17	Duodenum	23,288	7,913		89C-38	Duodenum	30,284	7,981
		Ileum	30,356	19,092			Ileum	45,147	26,992
		Jejunum	44,311	28,602			Jejunum	39,505	13,412
		caecum	59,151	47,817			caecum	65,810	54,713

NO. denotes number. 77T, 84T and 89T denote overfed group at day 7,14 and 19 of overfeeding while 89C denotes control group at day 19 of overfeeding; 77T-5, 6 and 7 are the liver samples from the overfed geese (treatment or T group) at 77 days of age (or at day 7 of overfeeding); the liver samples harvested at 84 and 89 days of age are similarly named. N=3 for each group.

Supplementary Table S12. The OTU classifications at phylum level from intestinal metagenome analysis (Average percentage)

Intestinal segment	Group	Firmicutes	Proteobacteria	Bacteroidetes	Cyanobacteria	Actinobacteria	Fusobacteria	Spirochaetes	Verrucomicrobia	Acidobacteria	Deferribacteres	others
Duodenum	77T	34.64	26.65	10.86	14.45	6.17	0.54	3.85	1.14	0.33	0.15	1.23
	84T	42.61	22.48	5.53	13.31	6.30	5.60	2.41	0.71	0.14	0.01	0.90
	90T	49.51	25.04	6.53	4.97	7.38	0.44	0.27	1.11	1.39	0.00	3.35
	90C	30.94	32.36	8.25	1.75	14.84	0.23	0.34	2.57	2.91	0.15	5.66
Jejunum	77T	32.19	30.61	5.13	20.03	8.42	0.40	0.26	0.72	0.67	0.01	1.56
	84T	55.78	20.22	7.66	8.28	2.11	0.17	1.89	0.66	0.96	0.01	2.25
	90T	45.63	22.49	10.11	2.27	9.09	0.22	0.31	1.65	2.63	0.01	5.58
	90C	33.46	25.48	7.54	2.48	12.21	0.21	0.34	2.96	4.18	0.15	11.01
Ileum	77T	31.62	38.55	3.42	18.23	6.16	0.27	0.18	0.54	0.17	0.05	0.82
	84T	58.56	35.02	1.82	0.64	2.71	0.23	0.23	0.32	0.08	0.00	0.38
	90T	45.53	25.31	5.60	9.45	7.01	0.17	0.16	1.62	1.24	0.02	3.89
	90C	44.65	30.25	8.59	1.20	8.80	0.73	0.28	1.36	1.25	0.21	2.68
Caecum	77T	25.38	15.77	28.69	0.34	0.10	11.19	13.29	3.68	0.02	0.60	0.94
	84T	12.10	17.64	14.45	0.17	0.22	44.18	9.19	0.36	0.05	0.07	1.57
	90T	14.97	42.39	19.89	2.05	4.94	0.30	0.12	5.44	3.25	0.00	6.64
	90C	27.96	25.63	12.37	1.80	0.42	0.25	8.81	6.13	0.13	8.70	7.79
Average		36.60	27.24	9.78	6.34	6.06	4.07	2.62	1.93	1.21	0.63	3.52

77T, 84T and 89T denote overfed group at day 7,14 and 19 of overfeeding, 89C denotes control group at day 19 of overfeeding. N=3 for each group.

Supplementary Table S13. The ratio of *Firmicutes* to *Bacteroidetes* in different intestinal segments of the overfed vs normally fed geese at different overfeeding time

	77T	84T	90T	90C	Average of 77T,84T and 89T
Duodenum	3.19	7.71	7.58	3.75	6.16
Jejunum	6.27	7.28	4.51	4.44	6.02
Ileum	9.25	32.18	8.13	5.20	16.52
Caecum	0.88	0.84	0.75	2.26	0.82
Average of duodenum, jejunum and ileum	6.24	15.72	6.74	4.46	

77T, 84T and 89T denote overfed group at day 7,14 and 19 of overfeeding, 89C denotes control group at day 19 of overfeeding. N=3 for each group.

Supplementary Table S14. The genus-level clustered taxonomic groups that identified from intestinal microbial metagenome analysis

See a separate supplementary file: Supplementary Table S14.xlsx

Supplementary Table S15. Information for intestinal microbes that differentially inhabited different intestinal segments

See a separate supplementary file: Supplementary Table S15.xlsx

Supplementary Table S16. Pathway analysis on the differentially expressed genes in the livers and predicted functional genes of gut microbiota in the intestines of the overfed geese vs normally fed geese

See a separate supplementary file: Supplementary Table S16.xlsx

Supplementary Table S17. Levels of SCFA in the sera of the overfed vs normally-fed geese [a]

	89C<br>(Mena ±Standard deviation)	89T<br>(Mean ± Standard deviation)	Fold change (89T over 89C)	P-value
**acetic acid**	**15073±3850.12** [b]	**16871.67±5930.18**	**1.12**	**0.55**
**propionic acid**	**196456.67±124271.84**	**188650.33±159663.64**	**0.96**	**0.93**
**isobutanoic acid**	**2455.83±1444.36**	**2665.67±832.99**	**1.09**	**0.76**
**butanoic acid**	**914.67±207.24**	**1306.67±225.6**	**1.43**	**0.01**

[a] SCFA denotes short chain fatty acid, n=6.

[b] The data indicate integral peak areas.

Supplementary Table S18. Detailed information on prediction of transcription factors of goose C5 and human C5

See a separate supplementary file: Supplementary Table S18.xlsx

Supplementary Table S19. Feed formula of the homemade feed used in the study

See a separate supplementary file: Supplementary Table S19.xlsx

Supplementary Table S20. The sequences of primers for quantitative real-time PCR

Primer name	sequence(5'to3')
C3 forward	TGGCGTTATGGTGGATGA
C3 reverse	CAGTGACGTAGAGCGAGTGTC
C4 forward	ACTGCTTACGCTCTGACGC
C4 reverse	CCACCGAGACTGCATTGTT
C5 forward	ACATTAGACCACCACGCTCCC
C5 reverse	CCGTTTGACAAGAAGGGAAT
TNFα forward	GTATGTGCAGCAACCCGTAG
TNFα reverse	TGGGCATTGCAATTTGGACA
HNF1α forward	AGAGCGTTCCCGTGATAAA
HNF1α reverse	AGGTTGGCGGTGTCTGTTA
GAPDH forward	GCCATCAATGATCCCTTCAT
GAPDH reverse	CTGGGGTCACGCTCCTG

Supplementary Methods

Metabolites Extraction and Sample Derivatization

One hundred microliter of thawed plasma sample, 50 μL of water, and 250 μL of 0.2mg/mL aqueous NaOH were added to a glass vial, followed by adding 250 μL of PrOH/pyridine solvent (v/v=3:2) and 50 μL of PCF to the vial. After a brief vortex, the derivatization reaction was carried out under ultra-sonication for 1 min. One hundred fifty microliter of hexane was then added to the reaction mixture, followed by 1 min of vortex and 10 min of centrifugation under 9000 g at 4 °C. The derivative extraction at upper hexane layer (150 μL) was transferred to a GC vial. The remaining solution was added with 100 μL of hexane. The procedure for vortex and centrifugation was performed with the mixture in the same way as before. One hundred microliter of supernatant was transferred and combined with the supernatant that was previously transferred. Ten milligram of anhydrous sodium sulfate was added to the supernatant to remove traces of water. A 50 μL of resultant mixture was vortexed prior to GC–MS analysis.

GC-MS Analysis

Metabolomics instrumental analysis was performed on an Agilent 7890A gas chromatography system coupled to an Agilent 5975C inert MSD system (Agilent Technologies Inc., CA, USA). A HP-5ms fused-silica capillary column (30 m × 0.25 mm × 0.25 μm; Agilent J&W Scientific, Folsom, CA) was utilized to separate the derivatives. Helium (>99.999%) was used as a carrier gas at a constant flow rate of 1 mL/min through the column. Injection volume was 1 μL in split mode with a ratio of 2:1, and the solvent delay time was 2.6 min. The initial oven temperature was held at 40 °C for 2 min, ramped to 70 °C at a rate of 10 °C/min, to 85 °C at a rate of 3 °C/min, to 290 °C at a rate of 41 °C/min, and finally held at 290 °C for 8 min. The temperatures of injector, transfer line, and electron impact ion source were set to 250 °C, 250 °C, and 230 °C, respectively. The impact energy was 70 eV, and data was collected in a sim mode (m/z 30-600).
